# Strain-Level Metagenomic Data Analysis of Enriched In Vitro and In Silico Spiked Food Samples: Paving the Way towards a Culture-Free Foodborne Outbreak Investigation Using STEC as a Case Study

**DOI:** 10.3390/ijms21165688

**Published:** 2020-08-08

**Authors:** Assia Saltykova, Florence E. Buytaers, Sarah Denayer, Bavo Verhaegen, Denis Piérard, Nancy H. C. Roosens, Kathleen Marchal, Sigrid C. J. De Keersmaecker

**Affiliations:** 1 Transversal Activities in Applied Genomics (TAG), Sciensano, 1050 Brussels, Belgium; Assia.Saltykova@sciensano.be (A.S.); Florence.Buytaers@sciensano.be (F.E.B.); Nancy.Roosens@sciensano.be (N.H.C.R.); 2 IDLab, Department of Information Technology, Ghent University, IMEC, 9052 Ghent, Belgium; Kathleen.Marchal@ugent.be; 3 National Reference Laboratory for Shiga Toxin-Producing Escherichia coli (NRL STEC), Foodborne Pathogens, Sciensano, 1050 Brussels, Belgium; Sarah.Denayer@sciensano.be (S.D.); Bavo.Verhaegen@sciensano.be (B.V.); 4 National Reference Center for Shiga Toxin-Producing Escherichia coli (NRC STEC), Department of Microbiology and Infection Control, Universitair Ziekenhuis Brussel (UZ Brussel), Vrije Universiteit Brussel (VUB), 1090 Brussels, Belgium; Denis.Pierard@uzbrussel.be; 5 Department of Plant Biotechnology and Bioinformatics, Ghent University, 9052 Ghent, Belgium; 6 Department of Genetics, University of Pretoria, Pretoria 0083, South Africa

**Keywords:** public health, foodborne outbreak investigation, strain-level metagenomics

## Abstract

Culture-independent diagnostics, such as metagenomic shotgun sequencing of food samples, could not only reduce the turnaround time of samples in an outbreak investigation, but also allow the detection of multi-species and multi-strain outbreaks. For successful foodborne outbreak investigation using a metagenomic approach, it is, however, necessary to bioinformatically separate the genomes of individual strains, including strains belonging to the same species, present in a microbial community, which has up until now not been demonstrated for this application. The current work shows the feasibility of strain-level metagenomics of enriched food matrix samples making use of data analysis tools that classify reads against a sequence database. It includes a brief comparison of two database-based read classification tools, Sigma and Sparse, using a mock community obtained by in vitro spiking minced meat with a Shiga toxin-producing *Escherichia coli* (STEC) isolate originating from a described outbreak. The more optimal tool Sigma was further evaluated using in silico simulated metagenomic data to explore the possibilities and limitations of this data analysis approach. The performed analysis allowed us to link the pathogenic strains from food samples to human isolates previously collected during the same outbreak, demonstrating that the metagenomic approach could be applied for the rapid source tracking of foodborne outbreaks. To our knowledge, this is the first study demonstrating a data analysis approach for detailed characterization and phylogenetic placement of multiple bacterial strains of one species from shotgun metagenomic WGS data of an enriched food sample.

## 1. Introduction

Whole-genome sequencing (WGS) is recognized as the most informative approach for subtyping of bacterial pathogens [1,2,3,4], and is currently increasingly being used in public health laboratories for surveillance and outbreak investigation of foodborne pathogens [1,4,5]. The standard WGS approaches are based on the use of bacterial isolates, which implies that multiple specific culturing steps, often preceded by an enrichment, need to be carried out in order to obtain a colony for sequencing [6,7]. The use of culture-independent diagnostics, such as metagenomic shotgun sequencing of clinical or food samples, could not only reduce the turnaround time of samples in an outbreak investigation, but also allow detection of multi-species and even multi-strain infections or outbreaks [8,9]. Moreover, isolation of a foodborne pathogen from a food matrix can be challenging because of low recovery of pathogenic isolates: a sample can be positive with qPCR screening, yet isolation of the detected strain may not be achieved [10].

While the ultimate goal of the metagenomic approach is to be able to identify pathogens from sequencing data of food products independently of any culturing steps at all, to link them to human isolates of affected persons, the application of this approach is limited by the very low number of pathogenic cells in many sample types. This limitation is currently being addressed by the use of a semi-selective enrichment step [11,12,13]. Several studies have emerged, exploring the use of metagenomic shotgun sequencing methods for detection of contaminating pathogens in enriched food samples [13,14,15], as well as mock communities [9], and fermented food products [16,17] which have a comparable composition as enriched food samples. These studies have provided a proof of concept for the feasibility of the metagenomic analysis of low complexity food microbiome data and tested different sample preparation and data analysis protocols. In Jarvis et al. [15] and Leonard et al. [13] the analysis of the obtained shotgun metagenomic data has been limited to identification of pathogens to species or phylogroup level. Many bacterial species are, however, opportunistic pathogens, with the pathogenic phenotype being specific to some strains, indicating that to fully use the potential of metagenomics in public health applications, strain-level data analysis needs to be carried out [18,19]. Leonard et al. [14] obtained strain-level phylogeny by assembling shotgun metagenomic data of a spinach sample spiked with a Shiga toxin-producing *Escherichia coli* (STEC), and taking the consensus single nucleotide polymorphisms (SNPs) in *E. coli* core genes to be the haplotypes (i.e., genetic determinants located on a single chromosome). However, for samples containing endogenous *E. coli*, the abundance of the endogenous strain caused the metagenomic sample to be placed outside the cluster containing the spiked STEC strain genome and in some cases even in a different phylogroup. These results underscored the need for methods to bioinformatically separate the genomes of two strains of the same species included in a microbial community, and hence present in the shotgun metagenomic data set. Walsh et al. [16] used a more advanced data analysis strategy based on a combination of marker genes and pan-genes based tools, i.e., MetaMLST [20], StrainPhlAn [21] and PanPhlAn [22]. PanPhlAn was also used in a later study by Walsh et al. [17]. These tools screen shotgun metagenomic sequencing data against databases of housekeeping genes (MetaMLST), species-specific marker genes (StrainPhlAn), and species-specific genes from the pangenome (PanPhlAn) to identify and functionally characterize the strains present in the samples based on short-read alignments. Despite having strain-level sensitivity, these approaches rely on an assumption that a single dominant strain of each species is present in the sample [20,21,23]. They, therefore, will not permit to characterize the pathogenic strain in case it is not the most abundant strain of the species in the metagenomic sample, and might predict chimeric strains resulting from the overlap of unrelated sequences in case of strains with comparable abundancies. Finally, in the work of [9], subspecies were identified by extracting reads of a pathogenic species using kraken2 [24] and determining the closest published reference genome using Mash [25]. That approach has been developed for samples containing one strain of each species, and will produce misleading output in case multiple strains from the same species co-exist in a sample.

Currently, a number of more advanced methods for the strain-level analysis of shotgun metagenomic samples have been developed. As an alternative to generating a simple consensus sequence of a reference genome, or profiling of marker genes, some tools attempt to reconstruct the haplotypes of all strains co-existing in a sample based on the frequencies of the observed polymorphisms (haplotype reconstruction-based tools, [22,26,27,28,29,30,31,32]). To achieve best performances, these approaches rely on a simultaneous analysis of a cohort of samples (as, e.g., human fecal sample series from healthy or diseased individuals [26,27,28,33], environmental sample series [29,34] and anaerobic digester metagenome time series [28]). If such a cohort is not available, the performance will depend strongly on the differences between relative abundances of the strains, as well as the coverage of the species of interest (e.g., >30× for Evorha [30]). Given current sequencing depths, such coverage levels may not always be reachable for enriched food samples where, despite the enrichment, a substantial fraction of the reads comes from the food matrix. This will especially be the case when several samples are multiplexed in a single sequencing run to save resources, to reduce costs and to increase throughput [12,13,14]. Moreover, such tools typically reconstruct only the core genome or a fraction thereof, which is sufficient to perform phylogenetic analysis and evolutionary studies, but does not permit to capture the full gene content of each strain, which is indispensable in an epidemiological context. Finally, one type of statistical methods disentangles co-existing strains in metagenomic samples by mapping sequence reads to reference assemblies in an extensive database, and modelling sample contents based on the observed read distribution [35,36,37,38]. While having an obvious limitation in case of poorly characterized species, such database-based approaches provide excellent results for well-characterized foodborne pathogens like *E. coli* that have thousands of sequenced genomes available. Some of these methods have also been demonstrated to detect strains represented by as few as several thousands of reads [36,38]. The database-based tools have currently been applied for the analysis of fecal [36,38] and archaeological [37] datasets, but to our knowledge not to food samples. We believe, however, that the aforementioned qualities make them an excellent candidate for this type of application. Moreover, tools of such type also classify the bacterial reads according to the strain of origin. This will make it possible, for each strain that is represented by a sufficient number of reads, to perform all downstream analyses in the same manner as it is done for the isolates. Among others, it will permit to obtain a high-resolution phylogenetic placement of co-existing bacterial strains from a single metagenomic sample, which is essential to robustly link clinical and foodborne isolates with epidemiological relevance.

The current work is a case study demonstrating the feasibility of strain-level metagenomics of enriched food matrix samples. This is accomplished by using a data analysis methodology whereby reads are classified against a reference genome database. It includes a brief comparison of two read classification tools, Sigma and Sparse, using a mock community generated by in vitro spiking of minced meat with a STEC isolate (*stx1, stx2, eae* positive) from a previous outbreak [39,40]. To further explore the possibilities and limitations of this data analysis strategy, the better performing tool was subsequently evaluated using in silico simulated metagenomic data. To the best of our knowledge, this is the first case study demonstrating the feasibility of detailed characterization and phylogenetic placement of multiple bacterial strains of one species based on shotgun metagenomic WGS data of an enriched food sample. The performed analysis allowed us to link the pathogenic strains from food samples to human isolates previously collected during the same outbreak, demonstrating that the metagenomic approach could be applied for the rapid source tracking of foodborne outbreaks. 

## 2. Results

### 2.1. Analysis of In-Vitro Spiked Metagenomic Samples

**Figure 1:**
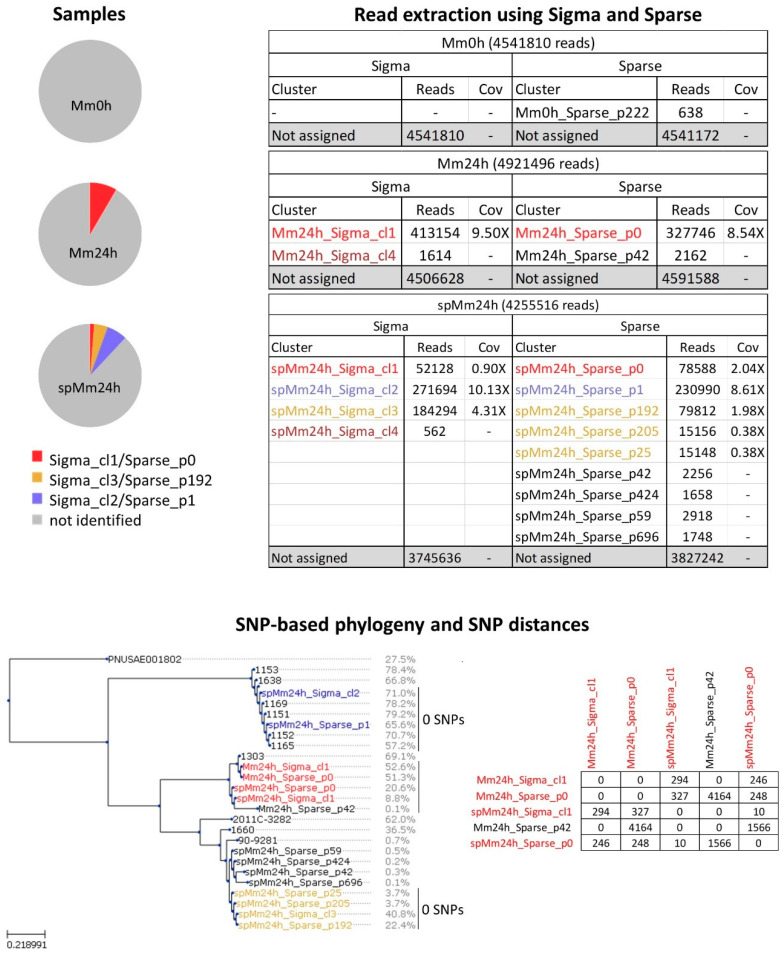
Strain-level metagenomic analysis of minced meat samples by Sigma and Sparse. Samples: the pie plots represent schematically the three metagenomic samples: the non-enriched minced meat sample containing no considerable endogenous *E. coli* strains (Mm0h), the enriched minced meat sample containing one more prevalent and one negligible (not included in figure) endogenous *E. coli* strain according to Sigma (Mm24h) and the minced meat sample spiked with isolate TIAC1152 and enriched containing three more prevalent and some negligible (not included in figure) *E. coli* strains, one of which (Sigma_cl2/Sparse_p1) corresponds to the spiked strain (spMm24h). Read extraction using Sigma and Sparse: the tables show Sigma and Sparse clusters detected in Mm0h, Mm24h and spMm24h, along with the corresponding number of reads and coverage (Cov). Single nucleotide polymorphism (SNP)-based phylogeny and SNP distances: on the left, SNP-based phylogeny of Sigma and Sparse clusters detected in metagenomic samples (colored) and some background isolates (black, Table 1) is shown. Percentages listed next to the Sigma and Sparse cluster names and isolate names represent the fraction of the reference genome that was suitable for the phylogenetic analysis. On the right, SNP distances (expressed as SNPs per million of genomic positions) observed within some of the groups of closely related strains and isolates are indicated. The colors of the Sigma and Sparse clusters correspond to the colors used in Figure S2 and Figure 2, allowing to identify the section of the reference genome cgMLST tree from which the references underlying the clusters originated.

Metagenomic sequencing data of the following samples were generated: minced beef meat (Mm0h), minced beef meat enriched for 24 h (Mm24h) and minced beef meat that was spiked with STEC isolate TIAC1152 at 5–10 colony forming units (CFU) per 25 g prior to enrichment for 24 h (spMm24h) (Figure 1). Mm0h and Mm24h were included to evaluate the background flora, including the presence of endogenous *E. coli* in the food matrix. Initially, the obtained data were analyzed with two different tools for the strain-level analysis of metagenomic samples, namely Sigma and Sparse (Figure 1 and Figure S1). 

Sigma showed that Mm0h contained no *E. coli* reads, while the two enriched samples, spMm24h and Mm24h, contained 11.9% and 8.4% *E. coli* reads, respectively. For spMm24h, nearly all reads were attributed to three separate clusters which accounted for 6.4%, 4.3% and 1.2% of the total number of reads (Figure 1, the clusters are referred to as “metagenomic sample name_read extraction tool_cluster in which the reads were retrieved”). These presumably corresponded to the spiked strain TIAC1152 as well as to two endogenous *E. coli* strains (spMm24h_Sigma_cl2, spMm24h_Sigma_cl3 and spMm24h_Sigma_cl1, Figure 1 and Figure S1). For Mm24h, the overwhelming majority of the *E. coli* reads originated from a single *E. coli* cluster (Mm24h_Sigma_cl1). In addition to the described large clusters, both spMm24h and Mm24h showed the presence of smaller clusters, spMm24h_Sigma_cl4 and Mm24h_Sigma_cl4, accounting for 0.01% and 0.03% of the reads, respectively. These clusters did not contain sufficient reads for phylogenetic analysis and harboured no serotyping nor virulence genes (see further). These results are generally concordant with the output of a qPCR analysis carried out prior to sequencing, which detected *E. coli* in both enriched samples (Mm24h and spMm24h), and indicated that the spMm24h contained a STEC.

The second tested tool, Sparse, found 638 *E. coli* reads in Mm0h, but no reads were assigned to average nucleotide identity (ANI) 99% clusters (~strain level, see Section 4). The number of reads recognized as *E. coli* and assigned to ANI 99% clusters for spMm24h and Mm24h were lower compared to Sigma, namely 10.1% and 6.7% of the total number of reads, respectively. For spMm24h, three larger ANI 99% clusters containing respectively 5.4%, 1.9% and 1.8% of the reads were observed (spMm24h_Sparse_p1, spMm24h_Sparse_p192 and spMm24h_Sparse_p0, Figure 1 and Figure S1), as well as several smaller ones (Figure 1 and Figure S1). Mm24h contained only one considerable ANI 99% cluster accounting for the majority of the bacterial reads, Mm24h_Sparse_p0, and a smaller cluster, Mm24h_Sparse_p42, accounting for 0.04% of the reads.

The reads attributed to the different reference genome clusters by Sigma or Sparse were extracted and used for phylogenetic analysis. The main clusters identified by Sigma and Sparse in spMm24h and Mm24h showed average coverages ranging between 10.13× and 0.90× if calculated with the STEC O157:H7 Sakai reference genome (Figure 1). For these, the percentage of the reference genome that was covered with a sufficient number of reads to be used for the phylogenetic analysis ranged from ~9% for spMm24h_Sigma_cl1, to 71% for spMm24h_Sigma_cl2 (Figure 1, SNP-based phylogeny and SNP distances). For comparison, these values were between 37% and 79% for the individually sequenced STEC isolates used as background in the phylogenetic tree construction (Figure 1, SNP-based phylogeny and SNP distances). spMm24h_Sigma_cl2 and spMm24h_Sparse_p1 showed 0 SNPs between each other, with the isolate TIAC1152 which was used for spiking, and with the other isolates from the same outbreak including the patient isolate, i.e., TIAC1169 (Figure 1, SNP-based phylogeny and SNP distances). The endogenous strains detected in the analyzed metagenomic samples by the two tools largely corresponded between each other, with 0 SNPs being detected between spMm24h_Sigma_cl3 and spMm24h_Sparse_p192 on the one hand, and Mm24h_Sparse_p0 and Mm24h_Sigma_cl1 on the other hand (Figure 1, SNP-based phylogeny and SNP distances). spMm24h_Sparse_p0 and spMm24h_Sigma_cl1 showed 10 SNPs between each other, showing that some differences in sequencing read assignment still existed between the tools. In addition, two of the smaller clusters identified by Sparse, spMm24h_Sparse_p205 and spMm24h_Sparse_p25, were grouped on the SNP-based phylogenetic tree and showed no SNP differences with spMm24h_Sigma_cl3 and spMm24h_Sparse_p192, indicating that they could contain reads belonging to the same strain as spMm24h_Sparse_p192. 

**Figure 2:**
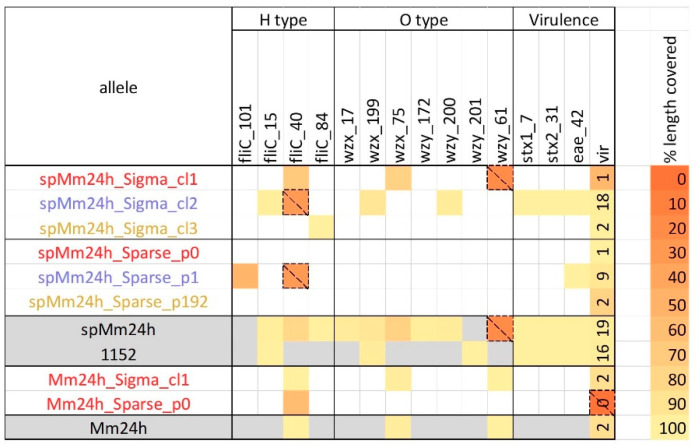
Gene detection of the O- and H-type serotyping and virulence genes performed on the clusters detected by Sigma and Sparse in the spiked (spMm24h) and unspiked (Mm24h) enriched minced meat samples. The table includes only the three largest clusters from spMm24h and only the first largest cluster from Mm24h, as none of the smaller clusters generated by any the two tools contained any of the monitored genes. In addition to the clusters generated by Sigma and Sparse, for comparison reasons, gene detection was performed on the reads obtained for the whole metagenomic samples, Mm24h and spMm24h, and on those of isolate TIAC1152 that was used for spiking. The Shiga toxin-producing *Escherichia coli* (STEC)-specific virulence genes (*stx* and *eae*), are displayed separately, while for the remaining virulence genes (vir), only the total number of the detected genes is shown (see Figure S3 for more detailed information). Cell color represents the percentage of the allele length covered by reads (%). Only alleles covered for more than 50% at least once are included in the table. Thereby, alleles that are covered below 50% are encased with dashed lines, and are not considered during interpretation of the results.

Further, virulence and serotyping gene detection were carried out. This is especially important, given the potential presence of different pathotypes of *E. coli* in food, characterized by their specific virulence gene content. STEC’s pathogenicity is linked to the production of Shiga toxins, encoded by *stx1* and/or *stx2*, in combination with an epithelial cell adhesion factor, encoded by the intimin gene (*eae*) [18]. Moreover, as a result of the acquisition and loss of multiple pathotype-defining virulence genes, a genotypic mosaic can emerge, of which *E. coli* O104:H4 causing the large 2011 German outbreak is a prominent example [41]. *E. coli* O157:H7 is the most common STEC serotype associated with outbreaks and severe diseases, although other STEC serotypes can occur [18]. In the whole spMm24h metagenomic sample, three alleles of the H antigen type, and five alleles of the O antigen type were detected (Figure 2). Most of these alleles were retrieved in the clusters generated by Sigma (Figure 2). Thereby, spMm24h_Sigma_cl2 contained the same H antigen type allele, and one of the two O antigen type alleles as the spiked isolate, while instead of the second O antigen type allele, wzy_201, another allele corresponding to the O157 type, wzy_200, was observed. Clusters produced by Sparse, on the contrary, contained none of the serotyping gene alleles detected in the whole spMm24h metagenomic sample nor of the spiked isolate. Similar results were observed for Mm24h (Figure 2). Interestingly, the endogenous strains from Mm24h and one of the main endogenous strains from spMm24h identified by Sigma, Mm24h_Sigma_cl1 and spMm24h_Sigma_cl1 respectively, shared two out of three serotyping gene alleles (Figure 2) and the majority of the reads belonging to these strains were attributed to the same reference genomes by Sigma (Figure S1), indicating that these strains were related to each other. The reference assembly to which the reads from both strains were mainly attributed, GCF_000829985.1, corresponds to *E. coli* strain 1303 isolated from a bovine mastitis sample (Table 1). Reads of the other main endogenous *E. coli* strain from the spMm24 background, represented by spMm24h_Sigma_cl3 were mainly attributed to the GCF_002741455.1 reference belonging to *E. coli* strain 90-9281, which is a pathogenic enterotoxigenic *E. coli* (ETEC). However, a directly neighbouring reference from the same reference genome cluster which also received some reads, GCF_001900675.1, belonged to a non-pathogenic *E. coli* strain D5 which has been isolated from dog faeces.

spMm24h_Sigma_cl2 harbored the same *stx* and *eae* gene alleles as the isolate TIAC1152, while the corresponding cluster detected by Sparse, spMm24h_Sparse_p1, did not contain any *stx* genes (Figure 2 and Figure S3). Besides the STEC-specific virulence genes (*eae* and *stx*), the whole spMm24h sample contained 19 additional virulence genes, out of which a few genes were represented by two alleles (Figure 2, spMm24h and Figure S3, spMm24h). Clusters identified by Sigma in spMm24h cumulatively contained 21 non-STEC-specific virulence genes, including all of the 19 non-STEC-specific virulence genes detected in spMm24h, with the majority of the genes (18) being observed in spMm24h_Sigma_cl2. In the clusters identified by Sparse, only 12 of the non-STEC-specific virulence genes were found, out of which, nine genes were in spMm24h_Sparse_p1 (Figure 2 and Figure S3). For both tools, a few genes in the extracted reads were represented by a different allele than observed in the whole spMm24h sample. These results indicate that the higher number of reads assigned by Sigma in each cluster resulted in a much higher retrieval rate of virulence genes compared to Sparse. A more detailed examination showed that some small discrepancies existed between the gene content of the isolate TIAC1152 and spMm24h_Sigma_cl2 (Figure 2 and Figure S3). These discrepancies can be explained by the fact that if only a fraction of a gene is covered by reads, this can interfere with the correct allele identification. As a result, a different allele can be detected based on data with different coverage or based on a potentially incomplete subset of reads retrieved from a metagenomic sample by Sigma. That newly identified allele can also have a shorter length than the original one, which in combination with the artificially set detection threshold, can result in the fact that a gene only becomes detected at a particular lower coverage.

### 2.2. In Silico Spiked Samples—Investigation of Different Coverage Ranges

**Figure 3:**
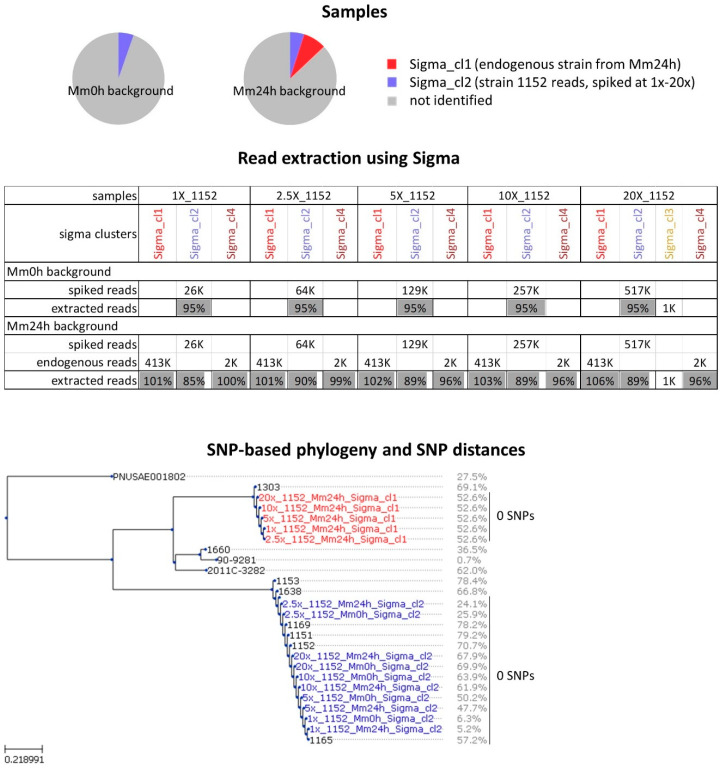
Strain-level analysis of in silico spiked metagenomic samples containing the strain TIAC1152 at different coverages. Samples: the pie plots represent schematically the simulated metagenomic samples consisting of isolate TIAC1152 reads down-sampled to different coverage and in silico spiked into the non-enriched minced meat sample containing no endogenous *E. coli* strains (Mm0h background) and the enriched minced meat sample containing one more prevalent and one negligible (not included in figure) endogenous *E. coli* strain according to Sigma (Mm24h background). Read extraction using Sigma: the number of isolate TIAC1152 reads surviving upon quality trimming spiked into the Mm0h and the Mm24h backgrounds (spiked reads), number of reads belonging to the endogenous strains from Mm24h according to Sigma (endogenous reads), and percentage of spiked and endogenous reads that were attributed by Sigma to clusters and extracted from the simulated metagenomic samples (extracted reads) are listed. For clusters containing in silico spiked reads, the percentage is calculated relative to the number of the in silico spiked reads. If the origin of the reads in a cluster is unclear, then the number of extracted reads is reported instead of a percentage. For clusters containing reads of the main endogenous strain from the Mm24h background, the percentage is calculated relative to the number of reads observed in the unspiked Mm24h sample. SNP-based phylogeny and SNP distances: SNP-based phylogeny of Sigma clusters detected in metagenomic samples and thus presumably corresponding to individual bacterial strains (colored) and some background isolates (black, Table 1) is shown. Percentages listed next to the Sigma cluster names and isolate names indicate the fraction of the reference genome that was suitable for the phylogenetic analysis. In addition, SNP distances (expressed as SNPs per million of genomic positions) observed within some of the groups of closely related strains and isolates are indicated.

Analysis of Mm24h and spMm24h showed that the approach for shotgun metagenomic data analysis that used Sigma permitted to discriminate, and at least partially characterize the individual bacterial strains contaminating the food matrix and performed better in comparison to the approach using Sparse. The analysis also allowed us to obtain a rough estimate of the coverage ranges that can be expected for the extracted reads of the individual strains within the in vitro enriched metagenomic samples. In order to estimate how accurately the reads are assigned to the different reference genomes by Sigma, and to what extent strains present at a different coverage could be characterized, additional tests were carried out using in silico simulated metagenomic data with the strain TIAC1152. Therefore, TIAC1152 isolate reads were downsampled to 1×, 2.5×, 5×, 10× and 20× and combined with the Mm0h and Mm24h reads, and the analysis was repeated as was done for the in vitro spiked samples (Figure 3).

For the samples in which Mm0h was used as background (i.e., no reads of the endogenous strain present), 95% of the in silico spiked reads were retrieved in a single Sigma cluster, while for the Mm24h-based samples, which already contained one main endogenous strain, only between 85% and 90% of reads belonging to the isolate TIAC1152 could be re-extracted (Figure 3, Read extraction using Sigma). On the other hand, the number of reads assigned to Mm24h_Sigma_cl1 (which corresponds to the main endogenous strain observed in Mm24h) was higher for the in silico spiked samples using Mm24h as background compared to the non-spiked Mm24h metagenomic sample (Figure 3, Read extraction using Sigma, extracted reads > 100%). This indicates that some of the spiked reads were miss-assigned to the cluster containing reads from the main endogenous strain.

The downstream analysis showed that for all coverages of spiked reads, including the lowest coverage (1×), the Sigma clusters corresponding to the spiked STEC strain TIAC1152 were direct neighbors of the TIAC1152 isolate on the phylogenetic tree, showing genetic distances of 0 SNPs between each other and with the outbreak isolates (Figure 3, SNP-based phylogeny and SNP distances). However, the phylogenetic placement becomes less reliable for lower coverages of spiked reads, this because the fraction of the reference positions that are covered by a sufficient number of reads to pass the quality filters for SNP calling becomes lower and a lower percentage of the genome can be reconstructed and used for phylogenetic analysis. More specifically, the percentage of the genome that could be used for phylogenetic analysis was for the 1× coverage only 5.2% with Mm24h and 6.3% with Mm0h as background (Figure 3, SNP-based phylogeny and SNP distances). However, starting from higher coverages this value sharply increases and reaches levels close to those observed for the reads obtained by sequencing the individual isolates if sufficient coverage is reached (here 5×–10×; Figure 3, SNP-based phylogeny and SNP distances). The Sigma clusters containing reads of the endogenous strain from the Mm24h background showed correct positions on the phylogenetic tree as well as distances of 0 SNP to each other (Figure 3, SNP-based phylogeny and SNP distances, all Mm24h_Sigma_cl1 clusters), this despite the fact that some of the strain TIAC1152 reads were miss-assigned to them. 

**Figure 4:**
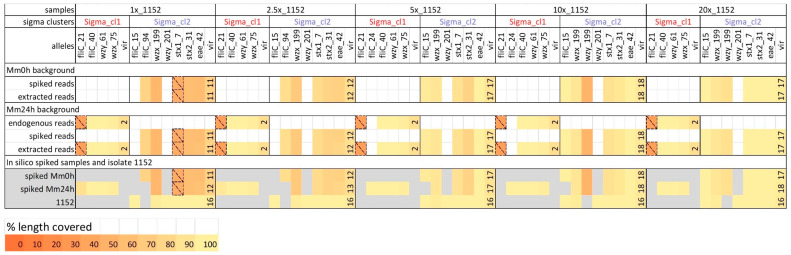
Strain-level analysis of in silico spiked metagenomic samples containing the strain TIAC1152 at different coverages: gene detection. Isolate TIAC1152 reads were down-sampled to different coverages (spiked reads) and spiked into the following metagenomic backgrounds: the non-enriched minced meat sample containing no endogenous *E. coli* strains (Mm0h background) and the enriched minced meat sample containing one more prevalent (endogenous reads) and one negligible endogenous *E. coli* strain according to Sigma (the latter strain contained no virulence or serotyping genes and is therefore omitted) (Mm24h background). The reads attributed to different Sigma clusters and thus presumably belonging to the different strains were extracted from the resulting in silico spiked metagenomic samples (extracted reads), and gene detection of the O- and H-type serotyping genes, STEC-specific virulence genes (*stx* and *eae*) and remaining virulence genes (vir) was performed. For the latter, only the total number of detected genes is shown (see Figure S4 for more details). The detected genes are grouped according to the Sigma clusters, in which the corresponding reads were retrieved. The line “endogenous reads” in the Mm24h background shows the genes observed in the main cluster extracted by Sigma from the unspiked Mm24h sample (Mm24h_Sigma_cl1). The lowest section of the table shows genes observed in the whole in silico spiked metagenomic samples prior to Sigma analysis (spiked Mm0h and spiked Mm24h) and in the non-downsampled sequencing data of isolate TIAC1152, the latter showing which serotyping and virulence gene alleles are expected for isolate TIAC1152. Cell color represents the percentage of the allele length covered by reads (%). Only alleles covered for more than 50% at least once are included in the table. Thereby, alleles that are covered below 50% are encased with dashed lines, and are not considered during interpretation of the results.

For the in silico spiked metagenomic samples generated using the Mm0h background, the same serotyping and virulence gene alleles were detectable in the spiked reads, the in silico spiked metagenomic samples (prior to Sigma analysis) and in the extracted reads belonging to the corresponding Sigma clusters (Figure 4 and Figure S4). For the in silico spiked metagenomic samples made with Mm24h as background, the presence of the additional genes of the endogenous strains in some cases interfered with the detection of the correct alleles in the in silico spiked metagenomic samples. The extracted reads, however, showed the presence of the same alleles as the spiked reads for all genes except one gene at 1× coverage. Moreover, despite the fact that some of the spiked reads were miss-assigned to the cluster containing reads from the endogenous strain from the Mm24h background, this did not affect the gene detection outcome for the endogenous strain (Figure 4 and Figure S4). For the H antigen type, the correct allele (*flic_15,* H7) was detected in the spiked and extracted isolate TIAC1152 reads at 20×, 10× and 5× coverages (Figure 4). At a lower coverage, another allele corresponding the H7 type (*flic_94*) was observed instead. For the O antigen type, one of the two expected alleles (*wzx_199*, O157) was detectable at all coverages, while the second expected allele (*wzy_201*, O157) was not detectable at any coverage. However, an alternative allele belonging to the O157 type (*wzy_199*) was observed at 10× and 20× (Figure 4). At 1× coverage, one of the two *stx* genes, the *eae* gene, and 11 other expected virulence genes were detectable (Figure 4 and Figure S4). Starting from 2.5× coverage, both *stx* genes and the *eae* gene were detected, and the number of other virulence genes increased to 12. At higher coverages, all virulence genes from isolate TIAC1152 were also observed in the spiked reads, although some of the genes were represented by a different allele (e.g., *espF*, *tccP* and *tir*, Figure S4). Thereby at 5×, all but one of the detected virulence genes were covered for over 70% of the gene length. It should be noted, however, that while isolate TIAC1152 harbored 16 virulence genes besides the *stx* and *eae* genes, spiked reads downsampled to 5×, 10× and 20× coverages contained one or two additional genes (Figure S4). Similarly to spMm24h_Sigma_cl2 discussed previously, these genes were *gad* for the 5× and 20× coverages, and *gad* and *iss* for the 10× coverage. The obtained results suggest that at a coverage of 5×, a sufficient fraction of the genome can be reconstructed to reliably link the strain to related isolates during the phylogenetic analysis, to identify strains that are pathogenic based on the presence of virulence genes, and to obtain a sufficiently complete virulence gene profile of the strain. 

### 2.3. In Silico Spiked Samples—Additional Test Cases

**Figure 5:**
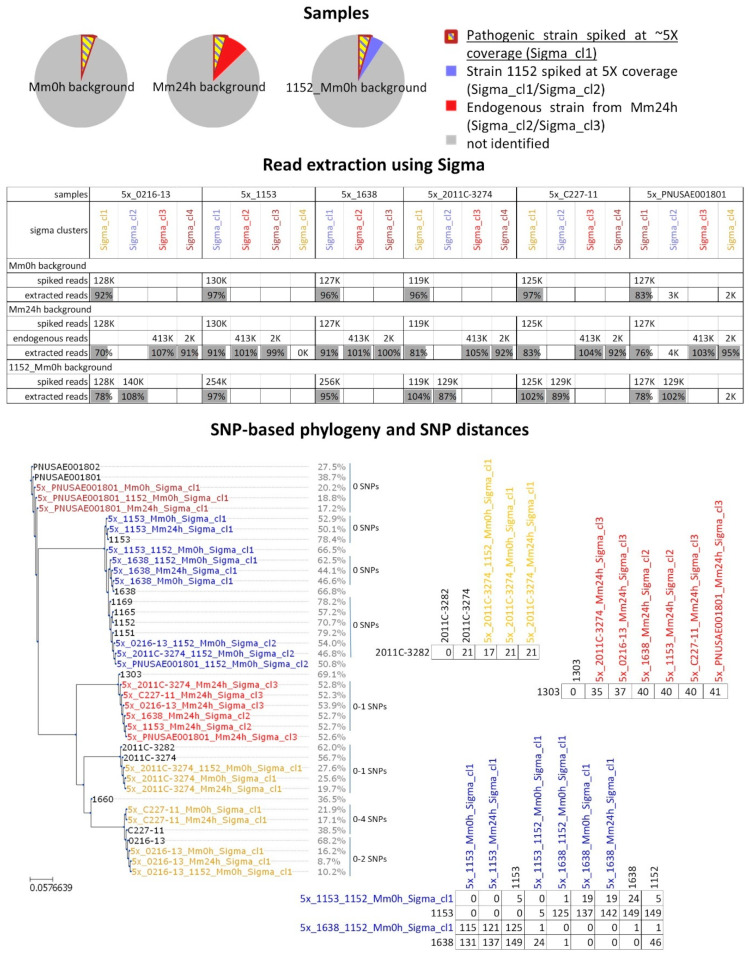
Strain-level analysis of in silico spiked metagenomic samples containing different pathogenic *E. coli* strains. Samples: the pie plots represent schematically the simulated metagenomic samples, consisting of reads of a pathogenic *E. coli* isolate (mixed color) in silico spiked at a ~5× coverage into the non-enriched minced meat sample containing no endogenous *E. coli* strains (Mm0h background), the enriched minced meat sample containing one more prevalent (red) and one negligible (not included in figure) endogenous *E. coli* strains according to Sigma (Mm24h background), and the non-enriched minced meat sample that has been previously in silico spiked with reads of a pathogenic *E. coli* isolate TIAC1152 (blue) at a coverage of 5× (1152_Mm0h background). Read extraction using Sigma: number of reads of a pathogenic *E. coli* isolate and isolate TIAC1152 surviving upon quality trimming spiked into the different backgrounds (spiked reads), the number of reads belonging to the endogenous strains of Mm24h according to Sigma (endogenous reads), and percentage of spiked and endogenous reads that were attributed by Sigma to clusters and extracted from the simulated metagenomic samples (extracted reads) are listed. For clusters containing in silico spiked reads, the percentage is calculated relative to the number of the in silico spiked reads. If the origin of the reads in a cluster is unclear, then the number of extracted reads is reported instead of a percentage. For clusters containing reads of the main endogenous strain from the Mm24h background, the percentage is calculated relative to the number of reads observed in the unspiked Mm24h sample. SNP-based phylogeny and SNP distances: on the left, SNP-based phylogeny of Sigma clusters detected in metagenomic samples and thus presumably corresponding to individual bacterial strains (colored) and some background isolates (black, Table 1) is shown. Percentages listed next to the Sigma cluster names and isolate names indicate the fraction of the reference genome that was suitable for the phylogenetic analysis. On the right, SNP distances (expressed as SNPs per million of genomic positions) observed within some of the groups of closely related strains and isolates are indicated.

All previous tests were carried out using only the strain TIAC1152 as the pathogenic *E. coli* strain, the same as used for the in vitro spiking experiment. In the current section, we evaluated the performance of the selected data analysis approach using artificial metagenomic samples mimicking single-strain contamination with other pathogenic *E. coli* strains (including different pathotypes). Hereto, the following six strains were used: two enteroaggregative *E. coli* (EAEC) strains related to the German O104:H4 outbreak [41], represented by isolates 0216-13 (O104:H4) and C227-11 (O104:H4); an enteropathogenic *E. coli* (EPEC) strain represented by isolate PNUSAE001801 (O167); and three STEC strains, represented by isolates TIAC1638 (O157:H7), TIAC1153 (O157:H7) and 2011C-3274 (O26:H11) (Table 1). The in silico spikes with these additional strains were created using the same amount of reads of the corresponding isolates as used to obtain 5× coverage for the TIAC1152 isolate and with Mm0h and Mm24h as background (Figure 5, Mm0h and Mm24h background). The actual resulting coverage was different for some of the samples because of a different read length, sequencing quality, and importantly the reference genome used, ranging between 1.2× for 0216-13 to 5× for TIAC1153.

Similarly to the strain TIAC1152, for the strains TIAC1153, TIAC1638, 0216-13, 2011C-3274 and C227-11 the spiked reads were assigned to a small number of relatively closely related reference assemblies, one of which was typically the most populated (Figure S5). For these strains, over 92% of reads could be retrieved in a single cluster from the Mm0h background (Figure 5, Read extraction using Sigma). For PNUSAE001801, however, the reads were equally shared between a somewhat larger number of assemblies all of which were more distinct from each other than in the previous cases (Figure S5). For that strain, the reads spiked into the Mm0h background were retrieved in three different clusters instead of a single one, with the largest cluster containing 83% of the original spiked data (Figure 5, Read extraction using Sigma). For all of the strains, the number of spiked reads retrieved from the more complex Mm24h background was between 5% and 22% lower compared to the Mm0h background (Figure 5, Read extraction using Sigma). The number of reads retrieved for the main endogenous *E. coli* strain present in Mm24h was higher than observed in the unspiked Mm24h sample.

Despite the somewhat lower retrieval rate of the spiked reads of the pathogenic *E. coli* strains from the Mm24h background compared to the Mm0h background, all of the extracted strains were placed in direct proximity of the corresponding isolate on the phylogenetic tree (Figure 5, SNP-based phylogeny and SNP distances). They differed by at most 2 SNP per million of genomic positions from the corresponding isolates, with the fraction of the reference genome that was suitable for the analysis ranging from 8.7% for 5×_0216-13_Mm24h_Sigma_cl1, to 52.9% for 5×_1153_Mm0h_Sigma_cl1 (Figure 5, SNP-based phylogeny and SNP distances).

In addition, distances of the extracted strains to some of the background isolates were evaluated (Figure 5, SNP-based phylogeny and SNP distances). Similarly to isolate PNUSAE001801 itself, 5×_PNUSAE001801_Mm0h_Sigma_cl1 and 5×_PNUSAE001801_Mm24h_Sigma_cl1 containing PNUSAE001801 reads extracted from the Mm0h and Mm24h background differed by 0 SNPs from the isolate PNUSAE001802, which belonged to the same outbreak according to NCBI pathogens. Both isolate 2011C-3274, and the clusters 5×_2011C-3274_Mm0h_Sigma_cl1 and 5×_2011C-3274_Mm24h_Sigma_cl1 containing 2011C-3274 reads extracted from Mm0h and Mm24h background, differed from isolate 2011C-3282 by 21 SNPs per million of genomic positions. The clusters containing reads of the main endogenous strain from the Mm24h background that were retrieved from the different in silico spiked samples all showed distances of 35-41 SNPs to the isolate 1303.

**Figure 6:**
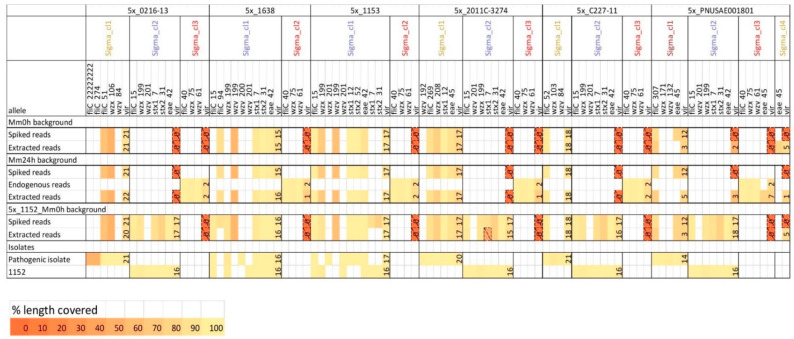
Strain-level analysis of in silico spiked metagenomic samples containing different pathogenic *E. coli* strains: gene detection. Reads from different pathogenic *E. coli* isolates (Table 1) were down-sampled to a coverage of ~5× (spiked reads) and in silico spiked into three metagenomic backgrounds: the non-enriched minced meat sample containing no endogenous *E. coli* strains (Mm0h background), the enriched minced meat sample containing one more prevalent (endogenous reads) and one negligible endogenous *E. coli* strain according to Sigma (the latter strain contained no virulence or serotyping genes and is therefore omitted) (Mm24h background), and an the non-enriched minced meat sample that has been previously in silico spiked with reads of a pathogenic *E. coli* isolate TIAC1152 (spiked reads, Sigma_cl2) at a coverage of 5× (1152_Mm0h background). The reads attributed to different Sigma clusters and thus presumably belonging to the different strains were extracted from the resulting in silico spiked metagenomic samples (extracted reads), and gene detection of the O- and H-type serotyping genes, STEC-specific virulence genes (*stx* and *eae*) and remaining virulence genes (vir) was performed. For the latter, only the total number of detected genes is shown (see Figure S6 for more details). The detected genes are grouped according to the Sigma clusters, in which the corresponding reads were retrieved. The line “endogenous reads” in the Mm24h background shows the genes observed in the main cluster extracted by Sigma from the unspiked Mm24h sample (Mm24h_Sigma_cl1). The last section of the table shows genes observed in the non-downsampled sequencing data of isolate TIAC1152 and the additional spiked pathogenic *E. coli* isolate (pathogenic isolate). Cell color represents the percentage of the allele length covered by reads (%). Only alleles covered for more than 50% at least once are included in the table. Thereby, alleles that are covered below 50% are encased with dashed lines, and are not considered during interpretation of the results.

All of the antigen type O and H alleles observed in the downsampled isolate reads that were used for spiking could be detected in the extracted reads belonging to the corresponding Sigma clusters (Figure 6, Mm0h and Mm24h background). However, the O and H antigen type alleles that were observed in the downsampled isolate reads used for spiking were in some cases different from those harbored by the original isolate, although corresponding to the same serotype, and in nearly all cases at least one of the expected alleles was not detectable in the downsampled isolate reads used for spiking (Figure 6, Spiked reads in Mm0h and Mm24h backgrounds versus Pathogenic isolate). In contrast, the *stx* and *eae* genes present in three additional pathogenic isolates, namely 2011C-3274, TIAC1638 and TIAC1153, were all detectable in the downsampled isolate reads used for spiking, and retrieved correctly by Sigma in all cases (Figure 6, Mm0h and Mm24h background). A large majority of the virulence genes that were present in the original isolates were also observed in the downsampled isolate reads, although sometimes presenting a different allele. Nearly all of the virulence genes that were detected in the downsampled isolate reads used for spiking were also retrieved in the extracted reads (=clusters) (Figure 6 and Figure S6). The retrieval of the virulence genes was not optimal only in case of PNUSAE001801, with a large number of genes being found in the cluster containing a small number of reads instead of the main cluster, that contained the PNUSAE001801 serotyping genes (Figure 6, Mm0h and Mm24h background, Figure S6). 

### 2.4. In Silico Spiked Samples—Mixed Samples

Finally, the method was evaluated using simulated metagenomic samples containing two pathogenic *E. coli* strains. The samples were generated using the Mm0h reads, which were spiked with isolate TIAC1152 reads at 5× coverage (TIAC1152_Mm0h background), and with reads of one of the pathogenic *E. coli* isolates described in the previous section spiked using the same amount of reads as used for isolate TIAC1152 (Figure 5, 1152_Mm0h background). 

As expected, the strains 0216-13, C227-11, 2011C-3274, and PNUSAE001801 were sufficiently distinct from the strain TIAC1152 to be retrieved in a separate cluster (Figure S5). For these four strains, over 78% of the reads could be recuperated from the metagenomic sample (Figure 5, read extraction using Sigma, 1152_Mm0h background), and the corresponding fraction of the reference genome covered was comparable to the values observed with the Mm24h samples in silico spiked with the same reads (Figure 5, SNP based phylogeny and SNP distances, Sigma_cl1 samples from the 1152_Mm0h background compared to Sigma_cl1 samples from the Mm24h background). For TIAC1152, the fraction of reads recovered varied within 15% of the value observed for 5x_1152_Mm0h_Sigma_cl2, which was 95% (Figure 5, Read extraction using Sigma, percentage of TIAC1152 reads extracted from the 1152_Mm0h background). 

Similarly to the in silico spiked metagenomic samples containing a single pathogenic strain, the Sigma clusters corresponding to all four strains were placed correctly on the phylogenetic tree, showing a distance of 0-1 SNPs per million of genomic positions to the original isolate, with only exception of 5x_C227-11_1152_Mm0h_Sigma_cl1 (the cluster containing C227-11 reads), which differed by 4 SNPs per million of genomic positions from isolate C227-11. Cluster 5x_2011C-3274_1152_Mm0h_Sigma_cl1 differed by 17 SNPs per million of genomic positions from isolate 2011C-3282 instead of 21 SNPs per million of genomic positions as observed between isolate 2011C-3282 and 5x_2011C-3274_Mm0h_Sigma_cl1, and isolate 2011C-3282 and 5x_2011C-3274_Mm24h_Sigma_cl1. The genetic distances between Sigma clusters containing TIAC1152 reads and the outbreak isolates were only affected if TIAC1152 was co-spiked with PNUSAE001801, where the distance between 5x_PNUSAE001801_1152_Mm0h_Sigma_cl2 and the outbreak isolates increased from 0 SNPs to 2-4 SNPs per million of genomic positions. For the simulated samples containing two pathogenic strains, retrieval of the virulence genes was almost as good as for the simulated samples containing a single pathogenic strain (Figure 6 and Figure S6). 

For the strains TIAC1153 and TIAC1638, the reads were assigned to the same group of references as for TIAC1152, making it impossible to separate the reads from the two co-spiked strains over different clusters (Figure S5). Thereby, the cluster containing reads of TIAC1152 and TIAC1638 (5x_1638_1152_Mm0h_Sigma_cl1), showed a distance of 1 SNP per million of genomic positions to both isolate TIAC1638 and isolate TIAC1152, while the distance between the isolates TIAC1638 and TIAC1152 consisted of 46 SNPs per million of genomic positions (Figure 5, SNP-based phylogeny and SNP distances), and between the corresponding clusters from Mm0h and Mm24h background—from 37 to 41 SNPs. Similarly, 5x_1153_1152_Mm0h_Sigma_cl1 showed a distance of 5 SNPs per million of genomic positions to isolates TIAC1153 and TIAC1152, which differed by 149 SNPs between each other (Figure 5, SNP-based phylogeny and SNP distances). For comparison, clusters containing reads of isolates TIAC1153 and TIAC1152 extracted from Mm0h and Mm24h background differed between each other by 126-146 SNPs per million of genomic positions. This effect could be expected, i.e., when SNP detection is performed on Sigma clusters containing reads of two different strains, the polymorphic positions that are only observed in one of the two strains show atypical allele frequencies, and are therefore removed by the applied minimal alternative allele frequency filtering (>95%). The position of Sigma clusters containing TIAC1152 and TIAC1153 reads on the phylogenetic tree was different from the positions of the isolates TIAC1152 and TIAC1153, while the Sigma cluster containing TIAC1152 and TIAC1638 reads was placed in proximity of isolate TIAC1638 (Figure 5, SNP-based phylogeny and SNP distances). Gene detection analysis showed that the presence of similar serotyping and virulence gene alleles in a metagenomic sample, as was the case for 5x_1153_1152_Mm0h and 5x_1638_1152_Mm0h, could interfere with the correct identification of alleles (Figure 6 and Figure S6).

## 3. Discussion

Shotgun metagenomics has successfully been used to detect bacterial strains contaminating food samples. The phylogenetic relationships of the bacterial strains could, however, not be identified unless the sample contained no additional strains of the same species [9,14] or unless the strain of interest was the most abundant for its species [16,17], which was not always the case for the samples tested. Results of these studies underscored the need for methods to bioinformatically separate the genomes of multiple strains of the same species included in a microbial community [14]. Currently, several types of bioinformatics data analysis tools exist, permitting to obtain a strain-level resolution of metagenomic samples, including haplotype-reconstruction-based tools and database-based tools, the latter being, to our opinion, the most suitable for the encompassed application. In the current study, we tested, using pathogenic *E. coli* as a case study, whether database-based read classification approaches can be used for processing metagenomic sequencing data of contaminated food samples, allowing to obtain the information necessary to perform foodborne outbreak investigation in public health settings. We used for this in vitro spiked minced meat samples (enriched and non-enriched), which already contained an endogenous *E. coli* population before spiking, and in silico spiked metagenomic samples.

Initially, two tools, Sigma [38] and Sparse [37] were tested on non-enriched (Mm0h) and enriched (Mm24h) minced meat samples, as well as an enriched minced meat sample which was spiked (spMm24h) with a STEC isolate (TIAC1152) previously obtained as a part of a foodborne outbreak investigation. As observed from the phylogenetic analysis, both of the tested tools identified the same main strains in the metagenomic samples. One of the strains found in spMm24h by each tool appeared to be identical to the spiked TIAC1152 isolate: the corresponding Sigma and Sparse clusters were grouped together with isolate TIAC1152, and with other isolates from the same outbreak, on the phylogenetic tree showing 0 SNPs between each other. Further, gene detection analysis demonstrated that the cluster extracted by Sigma (and hence corresponding to one genome) contained nearly the same set of virulence genes as the outbreak isolates, including *stx1*, *stx2* and *eae*, and the serotyping gene alleles, although varying, converged upon the same serotype as that of the outbreak isolates. The accurate description of the set of virulence genes in a strain can be important, especially for STEC, as the pathogenicity of the strain depends on the combination of different virulence factors in a single genome, as was illustrated during the Germany 2011 outbreak [47]. The corresponding clusters generated by Sparse contained fewer virulence genes, and none of the expected serotyping gene alleles, indicating that not all of the reads belonging to the spiked isolate were correctly identified. Because of the better performance, the subsequent analyses were carried out using Sigma only. The inferior results of Sparse could possibly be explained by the structure of the database, where each cluster is represented by only one sequence. This could have limited the correct assignment of the reads originating from the pan-genome, thereby negatively affecting gene detection analyses performed downstream. Additionally, the performance of Sparse could possibly be improved by adapting the running parameters of the tool. Nevertheless, the obtained results show that database-based tools are able to bioinformatically separate individual *E. coli* strains present in a shotgun metagenomic sample, allowing to perform the same downstream analyses on the detected clusters as would be performed on sequencing data of individual isolates. This implies that similarly to the current study, in a real outbreak investigation, it will be possible to determine whether there is a link between patient isolates, which are relatively easy to obtain as they occur in higher concentration in the infected person, and the *E. coli* strains present in the suspected food samples based solely on the shotgun metagenomic sequencing data of these food samples upon enrichment. This would allow source tracking without having to isolate the pathogen from the food sample, which is not always successful. Unlike the currently used approaches [9,14,16,17], this methodology can be applied irrespective of the presence of additional *E. coli* strains in the sample. Moreover, even in cases when human isolates are not (yet) available, the presented approach would permit to directly characterize the strains present in suspected food samples, including the detection of virulence genes and identification of serotypes that are typically associated with pathogenic strains, such as O157, and to determine phylogenetic relationships of the isolated strains with pathogenic strains isolated in the past. For strains for which no method existed yet for the detection in food, such as *E. coli* O104:H4 causing the large 2011 German outbreak, knowledge on serotyping and virulence genes, as well as other characteristics could help to establish rapidly a performant screening and detection/isolation method. This can be especially important for countries that do not have other than conventional methods available in their laboratory capacity.

Regarding the large variability of the coverages of the strains detected in the in vitro enriched samples, it was further necessary to determine to what extent a strain can be characterized based on sequencing data of a given coverage. Additional tests with reads from isolate TIAC1152 in silico spiked at a different average coverage into metagenomic backgrounds showed that a detailed description of a strain can be obtained at a coverage of 5×, at which the majority of the virulence genes could be detected for the strain TIAC1152, and ~50% of the reference genome was covered with a sufficient amount of reads to be used for phylogenetic analysis. Correct identification of serotyping gene alleles, and thus serotype prediction, required a higher coverage (such as 10× or 20×), but in a real-life situation, absence of this type of information can be compensated by the phylogenetic analysis results. Further, in silico spiking of isolate TIAC1152 reads showed that even at an average coverage of 1× (which has for example been encountered for one of the endogenous strains in the spiked sample), a sufficient fraction of the genome could be reconstructed to allow the correct placement of the Sigma clusters containing isolate TIAC1152 reads on the phylogenetic tree. This observation is concordant with other studies. For instance, Richter and Rosselló-Móra [48] have shown that ANI calculations have not been seriously affected by low coverage. They demonstrated that randomly sequencing 20% or more of the genome of query strains, or producing an alignment of 4% or more of their genome can serve phylogenetic purposes. Generally, these findings show that the coverages that were observed for the individual bacterial strains in metagenomic samples using the current methodology were sufficient for characterization of these strains. Moreover, while isolate TIAC1152 was spiked at as low as 5–10 CFU in 25 g of meat, the final average coverage of its genome in the sequencing data of spMm14h was the double of the coverage required for reliable gene detection and phylogenetic analyses (10× versus 5×). Therefore, a lower sequencing depth or shorter enrichment time could potentially be applied, implying lower sequencing costs or shorter sample processing times.

In addition to the in silico spiked metagenomic samples containing isolate TIAC1152 reads, the performance of Sigma was benchmarked using in silico spiked metagenomic samples containing reads of other pathogenic *E. coli* isolates, which were combined with minced meat reads with or without an endogenous *E. coli* strain. Based on the observations made with the coverage series of isolate TIAC1152 reads, the same number of reads was used for in silico spiking as was needed to obtain a coverage of 5× for isolate TIAC1152. Because of a different read length, quality of the reads and/or reference genome, the resulting coverage was, in some cases lower than 5×. While at such coverage, detection of correct serotyping gene alleles was less reliable, it was sufficient for the phylogenetic analysis. In all cases, it was possible to link the Sigma clusters with the corresponding isolates, and with the closely related isolates included in the phylogenetic analysis, illustrating that the performance of the method achieved with the strain TIAC1152, or STEC, was not a unique case. Moreover, the majority of the expected virulence genes were observed in the down-sampled reads used for spiking, and all of the virulence genes that were present in the reads used for spiking, were correctly retrieved in the corresponding clusters except for PNUSAE001801 (discussed below), allowing to clearly distinguish the pathogenic strains from the background endogenous strains.

As confirmed by a qPCR analysis, the food matrix already contained an endogenous *E. coli* population prior to spiking with the STEC isolate. From this food matrix, three different metagenomic samples were made and investigated, i.e., a non-enriched and non-spiked matrix, an enriched and non-spiked matrix and an enriched and spiked matrix. In the non-enriched and non-spiked matrix, no *E. coli* was found with Sigma. However, our analysis found in the spiked metagenomic sample two main endogenous strains, while in the enriched and non-spiked sample only one could be detected. The reason for this could be the incomplete homogenisation of the food matrix, in combination with the fact that during an enrichment, the different strains grow at different rates, and to a different final abundance [12,15,49]. These results are concordant with a qPCR analysis of the metagenomic samples, which detected *E. coli* in the enriched minced meat (Mm24h) and STEC in the spiked and enriched minced meat (spMm24h). The metagenomic analysis, however, yielded a more detailed description of the *E. coli* population in the samples, including the exact number of strains, the full range of virulence genes and the phylogenetic relationships of these strains at a much higher resolution. One strain from each sample showed a similarity between each other, being assigned to the same set of reference genome assemblies and containing the same serotyping gene alleles. Phylogenetic analysis indicated that these two strains showed moderate SNP distances between each other and to an *E. coli* strain 1303 which has been previously isolated from a bovine background [45]. In fact, 1303 is a mastitis-associated *E. coli* (MAEC) strain, which is known as a facultative and opportunistic pathogen recruited from the highly diverse bovine gastrointestinal microbiota [50]. The reads of the remaining considerable endogenous *E. coli* strain from the spiked sample were retrieved to a reference genome assembly of an ETEC [46], and to a lesser extent to a non-pathogenic *E. coli* strain D5 isolated from dog faeces (BioSample: SAMN03252423). The observed strain is, however, very unlikely to be a pathogen as it did not show the presence of any virulence genes with the database used. These results already indicated that the data analysis method is capable of discriminating multiple strains from the same species in a metagenomic sample. We extended this analysis by carrying out further testing with two pathogenic strains co-spiked in silico into a metagenomic background to mimic more complex situations. In case the co-spiked strains were sufficiently different from each other, the corresponding reads were retrieved in separate Sigma clusters, all of which could be placed correctly on the phylogenetic tree. In case of co-spiked pathogenic strains nearly all the virulence genes that were detectable in the spiked reads, were still present in the corresponding clusters for all strains except PNUSAE001801 (discussed below). The obtained results indicate that the tested data analysis approach can be used for food samples contaminated with multiple pathogenic strains, making it suitable for multi-strain outbreak investigation which are more difficult to tackle using the classical approaches. 

For strains that are not sufficiently different it was not possible to correctly separate the reads over the different reference genomes. As a consequence, when co-spiked with TIAC1152, strains TIAC1153 and TIAC1638, which belonged to the same serotype as TIAC1152, were retrieved in the same cluster as TIAC1152. Importantly, the fact that two strains were retrieved simultaneously in a single cluster did not affect the ability of the data analysis strategy to detect their relatedness to isolates: the Sigma clusters containing reads from two different strains showed small SNP distances to isolates representing both of these strains. Thus, in case of real-life samples that are simultaneously contaminated with multiple pathogenic strains that are not sufficiently distinct to be discriminated, it will still be possible to determine the relatedness of the detected cluster to patient isolates representing both strains. This would mean that it would be possible to do a source tracking of the outbreak. This would even still be better than when working with isolates in case of multi-strain outbreaks. Indeed, when isolation is done, and only one isolate is taken further in the characterization (because we may not know that another one, or more, are present in the sample, or when the other one(s) could not be isolated), this food isolate might not match with the patient’s isolate, leading to an unresolved outbreak. With the metagenomic approach, even with closely related strains for which the clusters are not separate, the source tracking could be done. While it is not expected to happen commonly, this limitation of the approach should, however, be taken into account when interpreting the results. For example, the endogenous strains detected in the analysed metagenomic samples could in fact consist of several strains which were not sufficiently different to be discriminated. The results of gene detection analysis can be less reliable in that case as the presence of multiple related serotyping or virulence gene alleles might interfere with the correct allele identification. In the future, additional tests could be performed to more precisely determine the limit of the presented data analysis strategy, e.g., in terms of an ANI value above which a good resolution between strains can be expected. However, for the application of source tracking (phylogeny), the data analysis approach is sufficient, as elaborated above. In case a complete resolution (characterization) of the strains is required, it can be achieved by haplotype reconstruction-based methods, given sufficient coverage of the strains of interest can be obtained, which can mean a higher sequencing cost, or possibly by long-read sequencing of the metagenomic sample.

An additional possibility for future improvement of the applied method was revealed during the current analysis, i.e., related to the actual database used. Reads belonging to TIAC1152, and to all except one of the additional tested strains were assigned by Sigma to well-resolved clusters of a limited number of reference assemblies, one of which typically received the majority of the reads, indicating that the database contained a reference genome that was sufficiently close to the strain under study to accommodate most of its reads. While some of the reads were miss-assigned between the clusters containing reads from the rather different pathogenic or endogenous strains, this did not affect the SNP- or gene detection, indicating that these reads most likely originated from genomic regions that were identical between the spiked and the endogenous strains. However, for one strain, more specifically PNUSAE001801, the reads were spread over a range of more distinctly related assemblies. PNUSAE001801 is a strain of the EPEC pathotype, which causes less severe symptoms compared to STEC, enterohaemorrhagic *E. coli* (EHEC) and EAEC, and has therefore received less attention from public health and scientific community. As a consequence, the database did not contain any reference genome of the same serotype (O167), or any other sufficiently close reference genome for that strain. Therefore, besides the main cluster corresponding to that strain, PNUSAE001801 reads contaminated several additional clusters, which resulted in a small alteration of the SNP distances for the co-spiked or endogenous strains. Some of the virulence genes were also retrieved in wrong clusters. Importantly, it was still possible to link the cluster containing PNUSAE001801 reads to isolate PNUSAE001802 originating from the same outbreak. This example, however, highlights the importance to maintain an as-complete-as-possible database for the species of interest, to ensure more reliable results. In the current analysis, we have made use only of complete reference assemblies available at NCBI. The use of all (complete and incomplete) reference assemblies available at RefSeq would permit to cover the diversity of *E. coli* in an even better manner, but could considerably increase the analysis time, especially given the high level of redundancy of the resulting database. Therefore the tested approach would benefit greatly from the creation of a curated, non-redundant database of reference genomes that evenly covers the diversity of the species of interest and that is maintained up to date. Moreover, the maintenance of up-to-date databases for major foodborne pathogens would benefit strongly from a common effort of public health and research institutions in sharing data, and this cross-sectors (i.e., human, environmental, animal, food). The availability of such ‘one health’ databases with whole-genome sequences of circulating isolates will also allow to increase the resolution of the phylogenetic tree, and hence the tracing of the origin of the food contamination. 

In conclusion, our analysis showed that the database-based approach for the strain-level analysis of metagenomic data permits to achieve bioinformatic separation of individual bacterial strains present in an enriched food sample. Similarly to sequencing data of individual isolates, the clusters extracted from the metagenomic data can be used for identification and characterization of the pathogenic strains. This was shown to be possible at a coverage which can be obtained at a reasonable cost. Moreover, in case sequence data of patient isolates are available, they can be linked to the pathogenic strain(s) from the causative food product in a high-resolution phylogenetic analysis, providing robust evidence for outbreak investigation and source tracking, even in case of multi-strain outbreaks. Thereby, unlike the methodologies previously applied for the metagenomic-based analysis of food samples, the presented data analysis strategy is suitable for samples that contain endogenous strains of *E. coli*. Moreover, the same approach can be used for other bacterial species for which a database with a sufficient number of reference genomes can be obtained. This paves the way for the use of metagenomics in foodborne outbreak investigation, eliminating the need to perform the laborious, time-consuming, and often unsuccessful culture-based isolation of the pathogen.

## 4. Materials and Methods

### 4.1. Sequencing Data

Sequencing data of isolates used in this study is summarised in Table 1. Isolate TIAC1152 was obtained from minced meat during an outbreak investigation, and was shown to be closely related to bacterial strains isolated from patients [39,40]. This isolate was used for in vitro spiking of a minced meat sample (spMm24h). The spike was prepared according to Barbau-Piednoir et al. [51] inoculating 5–10 CFU of the STEC isolate in 25 g of beef meat. The mix was manually homogenized in buffered peptone water (BPW) medium and incubated at 37 °C for 24 h. After the enrichment, 1 mL of the enriched broth was centrifuged at 6000× *g* for 10 min, and the cell pellets were stored at −20 °C until DNA extraction. Unspiked minced meat was incubated in the same conditions (Mm24h), or centrifuged directly without the incubation step (Mm0h) and stored as described for the spMm24h sample until DNA extraction. The DNA of the food matrices was extracted using Nucleospin Food (Macherey-Nagel, Düren, Germany) according to the manufacturer’s instructions. The presence of *E.coli* and STEC in the samples was verified using qPCR of the genes *uidA, eae, stx1* and *stx2* according to Barbau-Piednoir et al. [51]. The presence of *E.coli* was confirmed in the enriched minced meat and STEC was detected in the spiked minced meat. DNA libraries were prepared using the Nextera XT DNA Library Preparation Kit (Illumina, San Diego, USA) following the standard protocol, and sequenced on an Illumina MiSeq machine obtaining 250 bp paired-end reads. The number of reads obtained for each sample is specified in Figure 1. The metagenomic samples were generated during a series of experiments for the optimisation of metagenomic sample enrichment and extraction (BioProject PRJNA645436).

### 4.2. In Silico Spiking

Sequencing reads of isolates TIAC1152 (O:157:H7 STEC), TIAC1638 (O157:H7 STEC), TIAC1153 (O:157:H7 STEC), PNUSAE001801 (O167 EPEC), 2011C-3274 (O26:H11 STEC), C227-11 (O104:H4 EAEC) and 0216-13 (O104:H4 EAEC) were used for in silico spiking (accession numbers specified in Table 1). Hereto, reads of the TIAC1152 isolate were quality-trimmed with Trimmomatic 0.3 [52], and mapped on B00000007.3 reference genome which belongs to the same serogroup as the isolate using SMALT 0.7.6 [53]. The resulting coverage was determined using Qualimap 2.2.1 [54], and used to calculate the number of reads needed to obtain coverage of 1×, 2.5×, 5×, 10× and 20× (Figure 3, Read extraction using Sigma). Downsampling of the isolate sequencing data was performed with bbmap [55] function *reformat*. The resulting read subsets were combined with the Mm0h or Mm24h matrix reads to obtain in silico spiked metagenomic samples mimicking sequenced enriched food samples with (Mm0h) or without (Mm24h) endogenous *E. coli* strains, containing a STEC at different concentrations. For in silico spiking of the other *E. coli* isolates (Table 1), the same number of untrimmed reads was taken as used to obtain 5× coverage of TIAC1152 (Figure 5, Read extraction using Sigma). Generated read subsets were combined with Mm0h and Mm24h matrices to create additional test cases in which the food matrix, that does (Mm24h) or does not (Mm0h) contain endogenous *E. coli*, is contaminated with a pathogenic *E. coli* strain at approximately 5× coverage (the actual resulting coverage was different for some of the samples because of a different read length, and sequencing quality compared to TIAC1152 reads). Finally, Mm0h reads were in silico spiked with TIAC1152 reads at 5× coverage (1152_Mm0h) in combination with a second pathogenic *E. coli* strain at approximately 5× coverage to mimic a multiple strain contamination. The in silico simulated metagenomic samples are labelled by a combination of the label of the spiked strain (the prefix “TIAC” is omitted) along with its coverage (e.g., 5x_1152) and matrix name (e.g., Mm24h).

### 4.3. Metagenomic Analysis

Initially, two database-dependent tools for analysis of whole-genome sequencing data from metagenomic samples, Sigma [38] and Sparse [37] were tested. Both tools were applied at default settings using an in-house constructed database containing 728 complete reference genome of *Escherichia coli* available on NCBI at the time of the study. In cases when the database contains several closely related reference assemblies, or the strain in question is equally distinct from all assemblies included in the database, the reads of the strain will be attributed to a number of similar reference genomes instead of a single one. To circumvent this problem, the reference genomes in a Sparse database are grouped into clusters of genomes showing different levels of ANI. For the final analysis, ANI 99% clusters were used. Sigma does not provide this option. Therefore, for Sigma, a cgMLST tree of the 728 reference assemblies was generated (Figure S2). Therefore, the allelic profiles of the 2513 *E. coli* cgMLST genes were obtained for the reference assemblies using the method described in [40], upon which a phylogenetic tree was constructed using the ‘generate’ function from the Phylo module included in Biopython-1.72 [56], applying the neighbour-joining tree construction method. For each metagenomic sample, the numbers of reads attributed to each reference assembly were plotted on the generated tree, taking into account assemblies to which 0.05% or more reads identified as *E. coli* were assigned. The resulting phylogenies were examined to detect clusters of more closely related reference genomes that were populated with reads, pooling the reads per cluster for the downstream analyses (Figure S1). For Sparse, the read extraction statistics were calculated based on the observed read counts instead of the values reported by the tool, which did not always correspond. The clusters obtained by Sigma and Sparse from the metagenomic samples are further labelled with a combination of the metagenomic sample name (real, such as Mm24h, or simulated, such as 5x_1152_Mm24h), the read extraction tool (Sigma or Sparse), and the cluster in which the reads were retrieved (e.g., cl1 and cl2 for Sigma and p0 and p192 for Sparse).

### 4.4. Downstream Analysis

To test whether the extracted clusters could be used in the same type of downstream analyses as sequencing data of isolates, the SRST2 tool [57] was applied for detection of the following genes: *E. coli* virulence genes and *stx* genes using the dedicated VirulenceFinder databases [58], and O-antigen and flagellin genes (H-typing) using the dedicated SerotypeFinder databases [59], using a maximum divergence threshold of 10% and taking into account alleles covered for at least 50% of their length. STEC is defined as a pathogen by its capability to produce one of two types of Shiga toxins, coded in a bacteriophage containing the *stx1* or *stx2* genes [60]. The severity of the disease is amongst others predicted based on the presence of the *eae* gene, and monitoring of these genes is therefore suggested in the ISO/TS 13136:2012 [60]. Because of that, these three genes are reported separately from the remaining virulence genes. While the database of *E. coli* virulence genes allows the detection of the *stx* genes, the results obtained using the dedicated *stx* gene database are to our experience more precise and were therefore used for these genes. Besides the individual strains detected in metagenomic samples and isolates, gene detection was carried out on whole and in silico spiked metagenomic samples to provide an idea about their gene content. When interpreting these results it should be noted that precise identification of serotyping and virulence gene alleles in whole metagenomic samples containing several strains of the same or closely-related species is potentially prone to errors because of the presence of multiple homologous alleles of the same gene. The same is true for clusters that contained reads from two different strains (see further). In addition, phylogenetic analysis was performed by mapping the reads on the STEC O157:H7 Sakai reference genome (GenBank accession no. BA000007.3) using SMALT 0.7.6, calling SNPs using Samtools 1.9 [61] and filtering the SNPs with Bcftools 1.8 [62] to retain positions with a mapping quality above 50, a minimal allele frequency of 95% and a minimum depth of three reads. The detected SNPs were used for generation of a consensus genome using Bcftools 1.8. Thereby, the reference genome positions that did not pass the quality filters were masked. The percentage of the positions that passed the quality filters, and that could thus be reconstructed in the consensus genome, were calculated for each isolate and reported as additional information on the phylogenetic trees generated in the next step. Prior to the construction of phylogenetic trees, the consensus sequences were filtered to retain only informative positions with snp-sites 2.4.1 [63]. The phylogenies were inferred with RAxML 8.2.11 [64], using GTRGAMMA model, with 100 bootstrap replicates and performing a rapid bootstrap analysis and search for best-scoring ML tree in one program run. Sigma and Sparse clusters for which the fraction of the reference genome that was suitable for the phylogenetic analysis was below 0.1% were excluded from the phylogenetic tree. The SNP distances between each pair of isolates were calculated by counting the number of consensus genomic positions with unequal values, while ignoring positions with missing values, and reported as the number of SNPs observed per million of genomic positions that passed the quality filter for both isolates. The latter allowed to normalise the inter-isolate distances for isolates that were present at a lower coverage and for which a smaller fraction of the genome could be reconstructed. For isolate 90-9281 no Illumina sequencing data was available. Therefore, paired-end Illumina reads of 300 bp were simulated from the reference genome using randomreads.sh tool from bbmap to obtain a coverage of 100×.

## Figures and Tables

**Table 1:** Isolate sequencing data.

Isolate	Serotype	Pathotype	Additional Information	Reference	Accession Number
**TIAC1151**	**O157:H7**	**STEC**	**Beef meat, outbreak**	[39,40]	**SRR10201483**
**TIAC1152**	**O157:H7**	**STEC**	**Beef meat, outbreak**	[39,40]	**SRR10201465**
TIAC1153	O157:H7	STEC	Bovine Carcass swab, sporadic	[39,40]	SRR10201452
**TIAC1165**	**O157:H7**	**STEC**	**Human faeces, outbreak**	[39,40]	**SRR10201427**
**TIAC1169**	**O157:H7**	**STEC**	**Human faeces, outbreak**	[39,40]	**SRR10201416**
TIAC1638	O157:H7	STEC	Human faeces, sporadic	[39,40]	SRR10201408
TIAC1660	O113:H21	STEC	Human faeces, sporadic	[39,40]	SRR10201398
C227-11	O104:H4	EAEC	stx-negative O104:H4 strain	[42]	ERR883742
PNUSAE001801	O167	EPEC	PulseNet STEC genome reference library	PRJNA218110	SRR2982117
PNUSAE001802	O167	EPEC	PulseNet STEC genome reference library	PRJNA218110	SRR2982118
2011C-3282	O26:H11	STEC	PulseNet STEC genome reference library	[43]	SRR3360214
2011C-3274	O26:H11	STEC	PulseNet STEC genome reference library	[43]	SRR6373714
0216-13	O104:H4	EAEC	stx-negative O104:H4 strain	[44]	SRX522695
1303	O70:H32	MAEC	*E. coli* strain isolated from bovine mastitis	[45]	SRR3492218
90-9281	O128:H27	ETEC	Enterotoxigenic *E. coli* strain collected in 1988 in Bangladesh	[46]	NZ_CP024243.1

Isolates that were used for in silico spiking are underlined; isolates from the STEC outbreak described by Braeye et al. [39] and Nouws et al. [40] are highlighted in bold; the remaining isolates served as background for phylogenetic tree construction. For the ease of reading, the prefix “TIAC” is omitted from the names of isolates in figures, and from the names of Sigma clusters. EAEC: enteroaggregative *E. coli*, EPEC: enteropathogenic *E. coli*, ETEC: enterotoxigenic *E. coli*, MAEC: mastitis-associated *E. coli*, STEC: Shiga toxin-producing *E. coli.*

## Supplementary Materials

The following Supplementary Materials can be found at https://www.mdpi.com/1422-0067/21/16/5688/s1. Figure S1. Strain-level metagenomic analysis of enriched minced meat sample (Mm24h) and an enriched minced meat sample that has been spiked with a pathogenic *E. coli* isolate TIAC1152 (spMm24h) using Sigma and Sparse. Figure S2. Unrooted cgMLST phylogenetic tree of the 728 complete *E. coli* assemblies used in the reference genome databases of Sigma and Sparse. Figure S3. Detailed report of *E. coli* virulence gene detection performed on the clusters detected by Sigma and Sparse in the spiked (spMm24h) and unspiked (Mm24h) enriched minced meat samples. Figure S4. Detailed report of *E. coli* virulence gene detection performed on the in silico spiked metagenomic samples containing the strain TIAC1152 at different coverages. Figure S5. Strain-level analysis of in silico spiked metagenomic samples containing different pathogenic *E. coli* strains: Sigma. Figure S6. Detailed report of *E. coli* virulence gene detection performed on the in silico spiked metagenomic samples containing different pathogenic *E. coli* strains at a ~5x coverage.

## Abbreviations

ANI	Average Nucleotide Identity
CFU	Colony-Forming Unit
cgMLST	core genome Multilocus Sequence Typing
EAEC	Enteroaggregative *E. coli*
EHEC	Enterohaemorrhagic *E. coli*
EPEC	Enteropathogenic *E. coli*
ETEC	Enterotoxigenic *E. coli*
MAEC	Mastitis-associated *E. coli*
Mm0h	Minced Meat sample enriched for 0 h
Mm24h	Minced Meat sample enriched for 24 h
SNP	Single Nucleotide Polymorphism
spMm24h	spiked Minced Meat sample enriched for 24 h
WGS	Whole Genome Sequencing
