# Supplementary material for: Strain-Level Metagenomic Data Analysis of Enriched In Vitro and In Silico Spiked Food Samples: Paving the Way towards a Culture-Free Foodborne Outbreak Investigation Using STEC as a Case Study
Source: Int J Mol Sci. 2020 Aug 8;21(16):5688. doi: 10.3390/ijms21165688 (PMC7460976; doi:10.3390/ijms21165688)
Supplement: Supplementary file 1 [file ijms-21-05688-s001.zip › ijms-882198supplementary/Supplementary_Figure_1.docx]

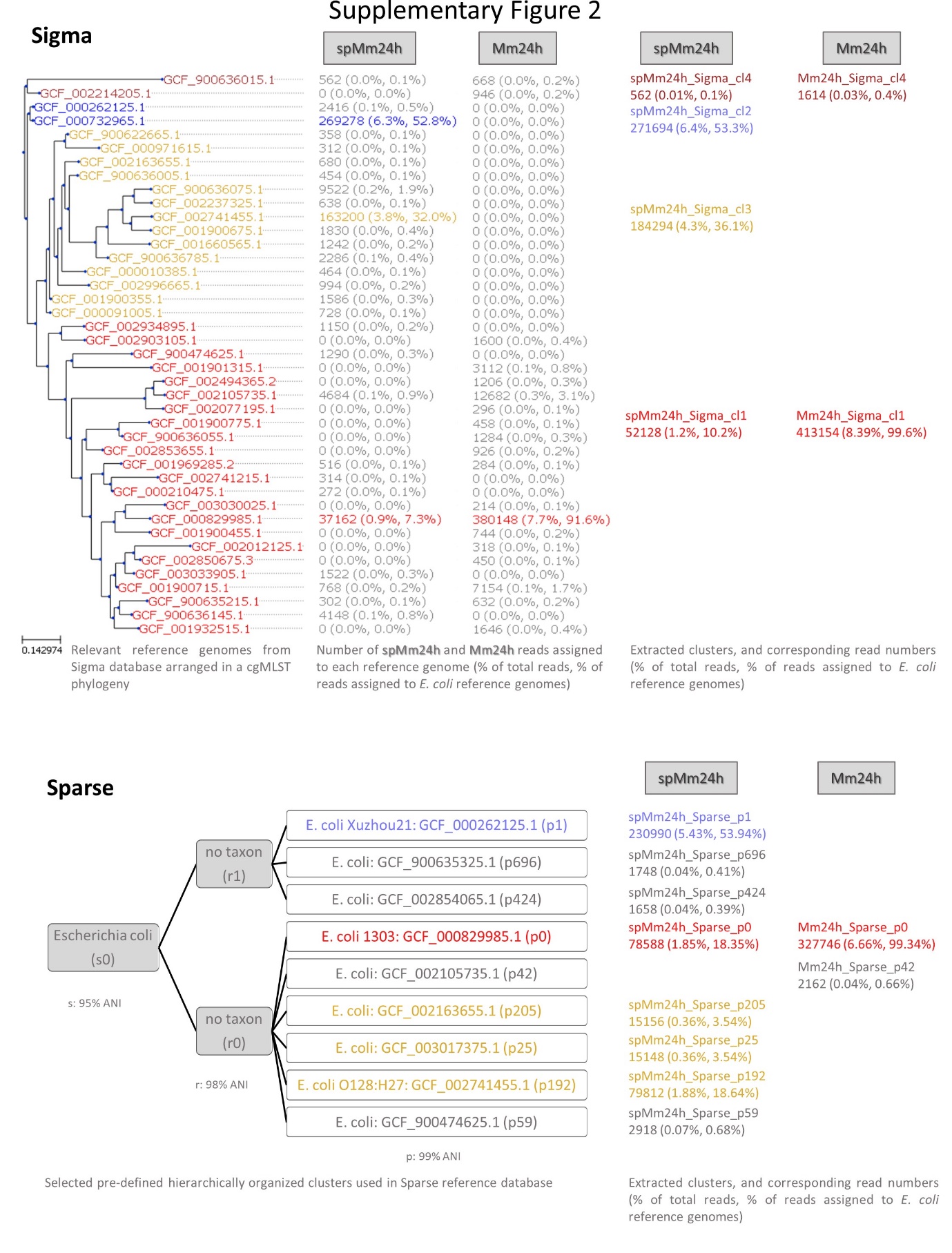
**Supplementary Figure 2. Strain-level metagenomic analysis of enriched minced meat sample (Mm24h) and an enriched minced meat sample that has been spiked with a pathogenic *E. coli* isolate TIAC1152 (spMm24h) using Sigma and Sparse.** Above: number of reads from Mm24h and spMm24h samples assigned by Sigma to each reference genome from the database containing 728 complete *E. coli* genomes. A cgMLST phylogeny of the reference genomes to which at least 0.05% of *E. coli* reads were assigned for any of the two processed samples is shown on the left. In the middle, the number of Mm24h and spMm24h reads assigned to each reference genome are listed. Reads from groups of closely related reference genomes were pooled to generate the clusters listed on the right. The colors of the references and of the clusters correspond to the colors used in **Supplementary Figure 1**, allowing to identify the section of the reference genome cgMLST tree from which the underlying references originated. Below: number of reads from Mm24h and spMm24h assigned by Sparse to each cluster from the database of 728 hierarchically ordered complete *E. coli* genomes. The clusters of assemblies with an average nucleotide identity (ANI) of 95%, 98% and 99% to which any *E. coli* reads were assigned for any of the two processed samples are shown on the left. Resulting clusters are shown on the right. The Sparse clusters containing a considerable amount of reads carry the same color as the corresponding Sigma clusters (see also **Figure 1**).
