# Supplementary material for: Strain-Level Metagenomic Data Analysis of Enriched In Vitro and In Silico Spiked Food Samples: Paving the Way towards a Culture-Free Foodborne Outbreak Investigation Using STEC as a Case Study
Source: Int J Mol Sci. 2020 Aug 8;21(16):5688. doi: 10.3390/ijms21165688 (PMC7460976; doi:10.3390/ijms21165688)

**Supplementary Figure 1.** **Unrooted cgMLST phylogenetic tree of the 728 complete *E. coli* assemblies used in the reference genome databases of Sigma and Sparse.** The colors used to mark the different sections of the tree correspond to the colors of the reference genome clusters used in all figures and tables, allowing identify the section of the tree harbouring the assemblies associated with each cluster.


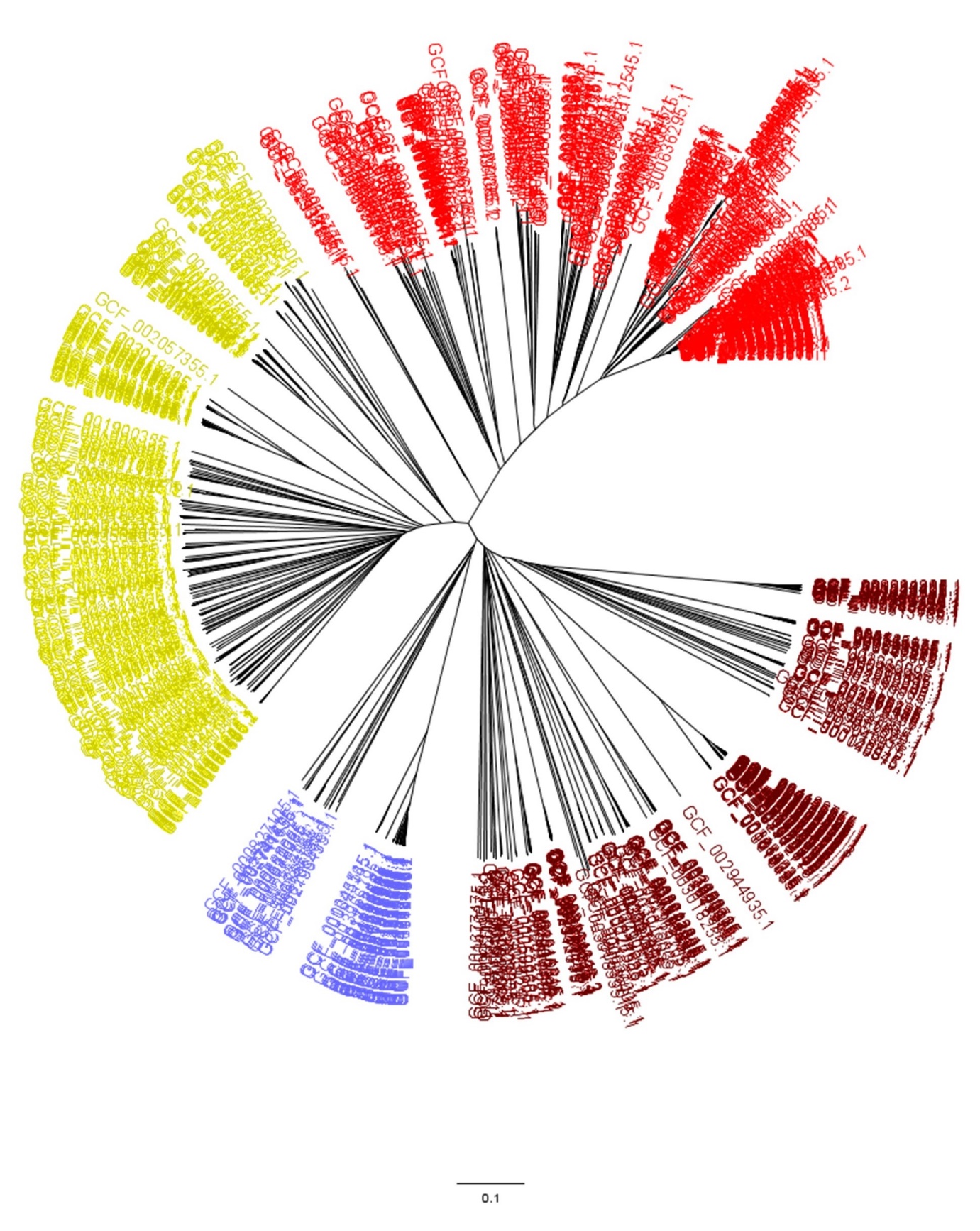

Supplement: Supplementary file 1 [file ijms-21-05688-s001.zip › ijms-882198supplementary/Supplementary_Figure_2.docx]
