# Supplementary material for: Strain-Level Metagenomic Data Analysis of Enriched In Vitro and In Silico Spiked Food Samples: Paving the Way towards a Culture-Free Foodborne Outbreak Investigation Using STEC as a Case Study
Source: Int J Mol Sci. 2020 Aug 8;21(16):5688. doi: 10.3390/ijms21165688 (PMC7460976; doi:10.3390/ijms21165688)
Supplement: Supplementary file 1 [file ijms-21-05688-s001.zip › ijms-882198supplementary/Supplementary_Figure_5.docx]

Continued on the next page


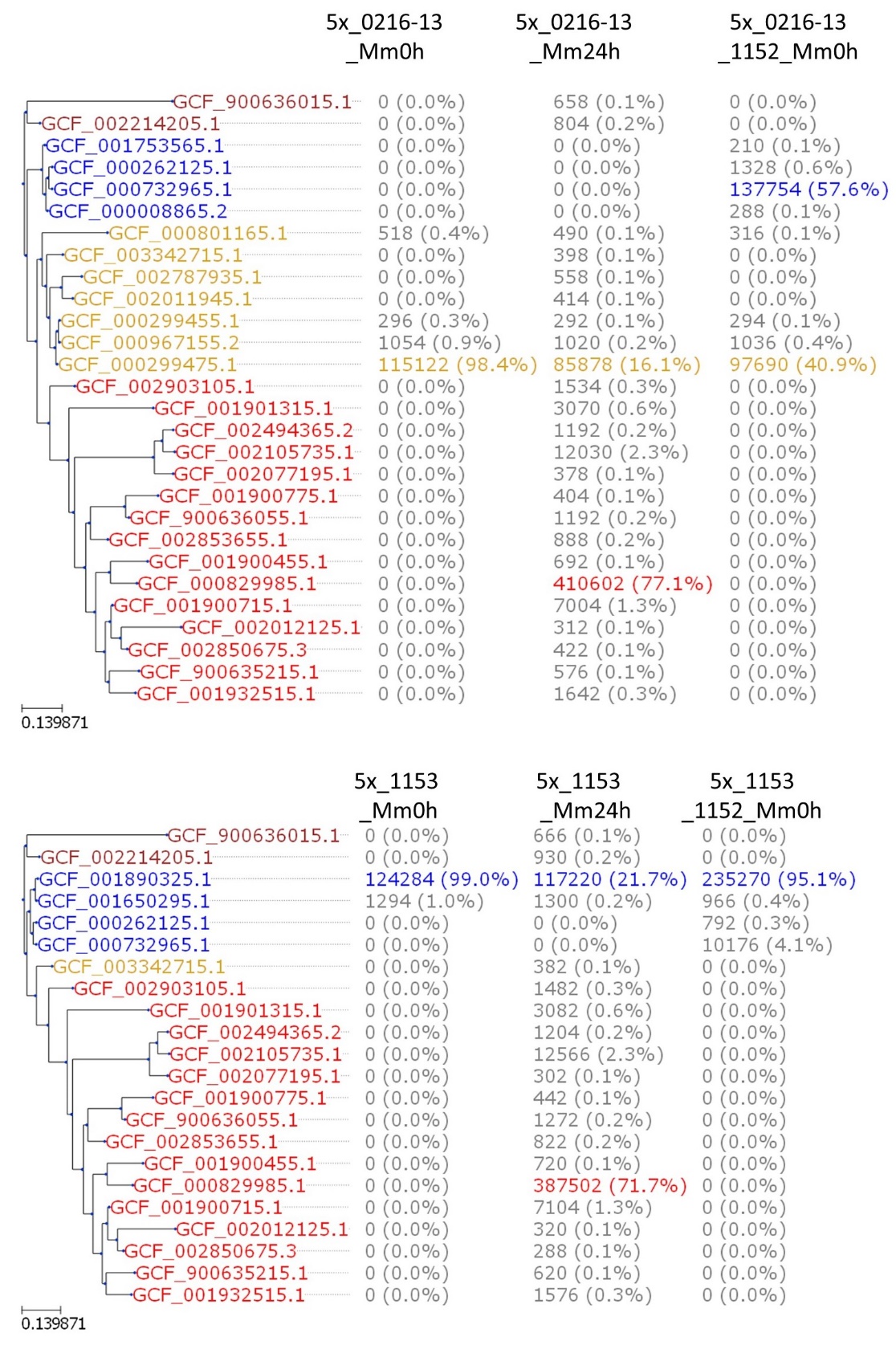


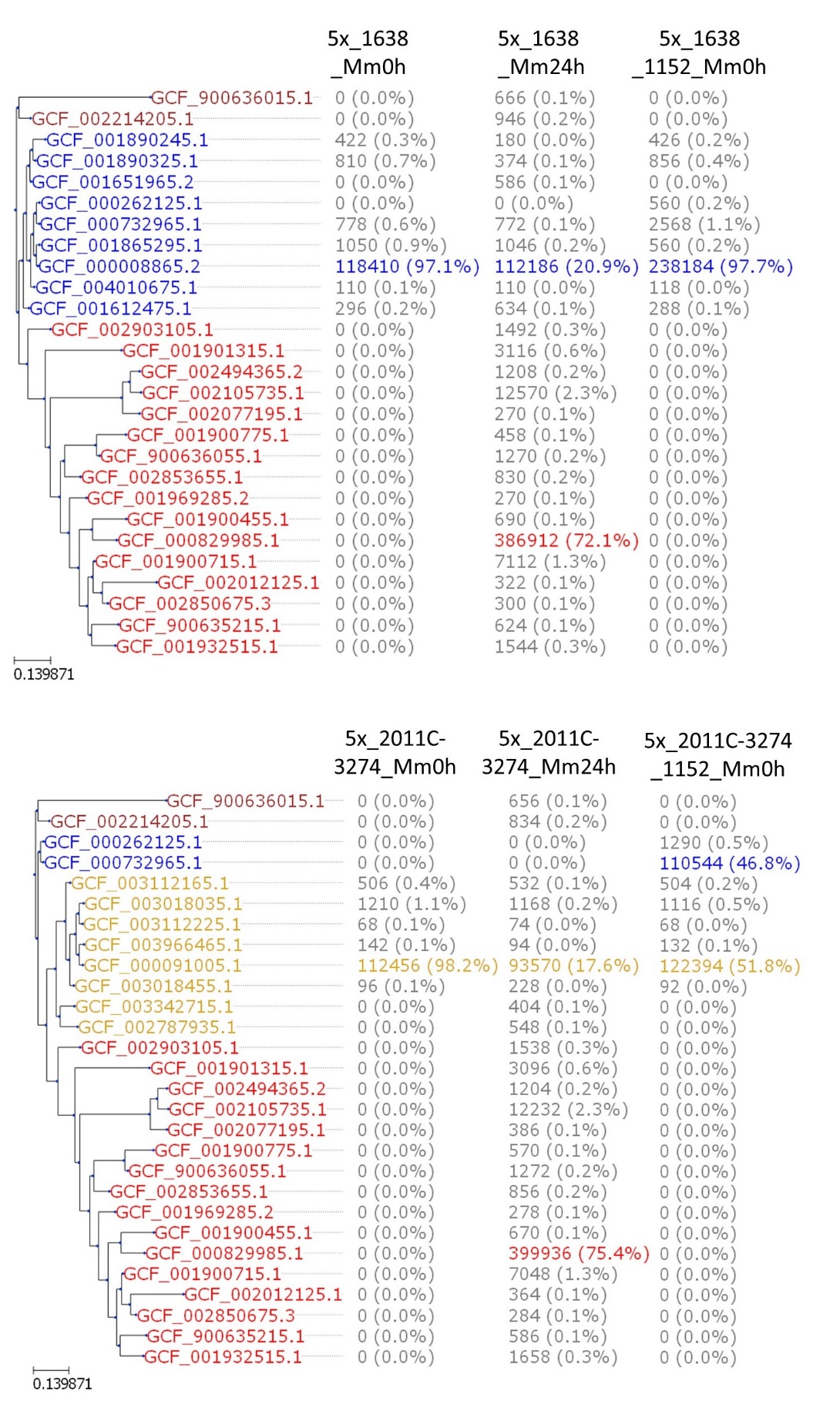


Continued on the next page


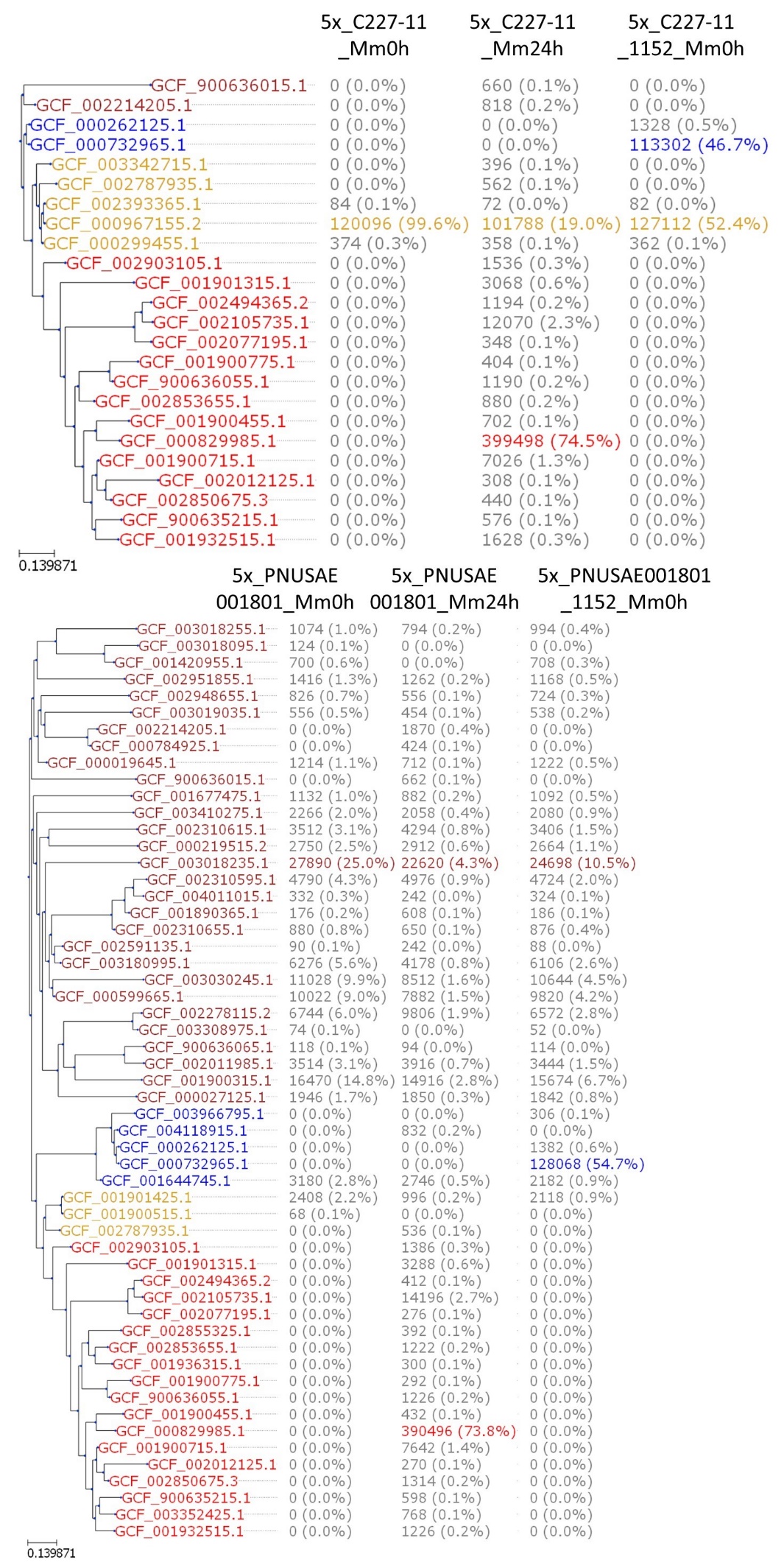


**Supplementary Figure 5. Strain-level analysis of *in silico* spiked metagenomic samples containing different pathogenic *E. coli* strains: Sigma**. The metagenomic samples that were spiked are: the non-enriched minced meat sample containing no endogenous *E. coli* strains (Mm0h background), the enriched minced meat sample containing one more prevalent (red) and one negligible (brown) endogenous *E. coli* strains according to Sigma (Mm24h background), and the non-enriched minced meat sample that has been previously *in silico* spiked with reads of a pathogenic *E. coli* isolate TIAC1152 (blue) at a coverage of 5X (1152_Mm0h background). These samples have been spiked with reads of six different pathogenic *E. coli* isolates downsampled to the same amount as used to achieve 5X coverage for isolate TIAC1152. For each isolate, a truncated cgMLST phylogenetic tree, containing references genomes to which at least 0.05% of *E. coli* reads were assigned for any of the processed samples, is shown on the left. In the middle, the number of reads, and the percentage of the total number of assigned reads, assigned to each reference assembly are listed for the three *in silico* spiked samples. Reads from clusters of closely related reference assemblies were pooled to generate the clusters shown on the right. The colors of the assemblies correspond to the colors used in **Supplementary Figure 1**, allowing identify the section of the reference genome cgMLST tree from which the references originated.
